# Mitochondrial DNA revealed the validation of *Quasipaa robertingeri* (Amphibia: Anura: Dicroglossidae) and its population genetic diversity

**DOI:** 10.1080/23802359.2021.1881836

**Published:** 2021-03-01

**Authors:** Gang Wang, Simeng Du, Gang Wei, Bin Wang, Shize Li, Ningning Lu

**Affiliations:** a College of Chemistry and Life Sciences, Chengdu Normal University, Chengdu, China; b Chengdu Institute of Biology, Chinese Academy of Sciences, Chengdu, China; c University of Chinese Academy of Sciences, Beijing, China; d Biodiversity Conservation Key Laboratory, Guiyang College, Guiyang, China; e Department of Food Science and Engineering, Moutai Institute, Renhuai, China

**Keywords:** Phylogenetic relationships, genetic diversity, clade, frog

## Abstract

The spiny frog *Quasipaa robertingeri* is endemic to a narrow region of southwest China and its taxonomic validation is still controversial. Based on COI gene sequences of 110 individuals from seven populations of *Q. robertingeri* and its related species, we investigated the phylogenetic position and population genetic structure of the species. Phylogenetic analyses indicated that *Q. robertingeri* was deeply genetically separated from its closely related species *Q. boulengeri*, indicating the validation of the species. All samples of *Q. robertingeri* were clustered into two divergent lineages. Haplotype network, AMOVA, and genetic distance estimations also supported the separations of the two groups. Neutrality tests indicated that one lineage has been likely independently experienced a recent population expansion, leading to a secondary contact area between the two lineages.

## Introduction

The spiny frog *Quasipaa robertingeri* belongs to the family Dicroglossidae (Amphibia, Anura; Frost 2020), and is commonly found in the mountain streams at elevations ca. 400–2000 m in a narrow region, bordering Sichuan and Guizhou provinces and Chongqing City in southwest China (Fei et al. 2009, 2012). The validation of the species is still on debates. Some molecular phylogenetic studies suggested that it should be synonym with *Q. boulengeri*, which is broadly sympatric with *Q. robertingeri* (Che et al. 2009; Zhang et al. 2018). But Fei et al. (2009, 2012) still recognized *Q. robertingeri* as a valid species based on morphology. Whatever, there has been no work with comprehensive sampling on the population level of the species and its related species to resolve the systematic problems.

In recent years, a variety of human-caused threats especially excessive captures and habitat destructions lead to the dramatic decline of *Q. robertingeri* (Jiang et al. 2016). In view of the serious threats and their high sensitivity to environmental factors, this species was listed as the VU (vulnerable) species in the red list of China’s vertebrates (Jiang et al. 2016). However, knowledges especially on the population diversification of this species remains scarce, and it is obligatory for us to put strategies to resolve the status of the species. Investigations on population diversification of the VU species would promote conservation of its endemic germplasm resources and genetic diversity.

In light of its rapid rate of evolution and maternal inheritance (Sun et al. 2012), mitochondrial DNA markers have been often used to evaluate genetic diversity, detect phylogenetic relationships, and recognize phylogeographic clusters (Weiss et al. 2011; Liu et al. 2015; Wang et al. 2017). In this study, the mitochondrial cytochrome oxidase subunit I (COI) gene was used to investigate phylogenetic position of *Q. robertingeri* and its population genetic diversity.

## Materials and methods

**Figure 1.**
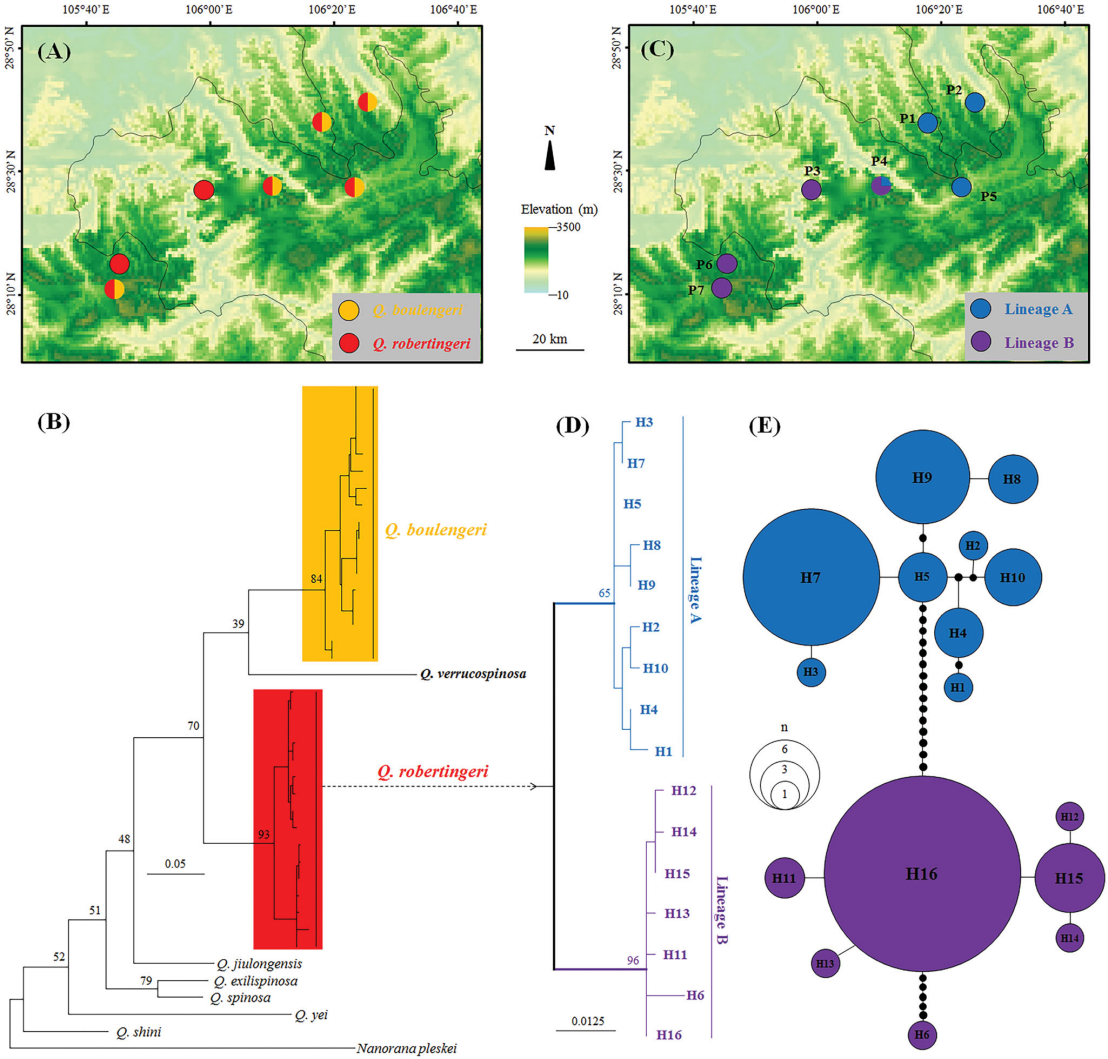
Sampling localities in this study and phylogenetic relationships of *Quasipaa robertingeri*. (A) Sampling localities in this study, showing sympatric distributions of *Q. robertingeri* and *Q. boulengeri*. (B) Maximum-likelihood tree based on COI gene sequences of *Q. robertingeri* and its congeners. Bootstrap support values were denoted near nodes. (C) Sampling localities in this study, showing distributions of lineages of *Q. robertingeri*. (D) Phylogenetic relationships of haplotypes of *Q. robertingeri*. (E) Haplotype network of *Q. robertingeri*. The circle size is proportional to the number of samples. One black dot means one mutation.

A total of 110 specimens of *Q. robertingeri* were collected from seven localities (P1–P7) scattered in the boundary of Sichuan and Guizhou provinces and Chongqing City, China (Figure 1(A,B); Table 1). In addition, 10 specimens of *Q. boulengeri* were also collected in six same places together with *Q. robertingeri* (Figure 1(A)). Tissues were collected and stored separately in 95% ethanol before specimen fixation. Specimens were preserved in the Chengdu Institute of Biology, Chinese Academy of Sciences (CIB, CAS). Total DNA was extracted from the tissues using a standard phenol–chloroform extraction procedure (Sambrook et al. 1989). COI primers and PCR protocols follow Che et al. (2012). Sequencing was conducted in both directions using the same primers as used in the PCR using ABI3730 sequencer in Shanghai Generay Biotech Co. Ltd. (Shanghai, China). The new sequences were deposited in the GenBank with accession numbers MW143300–MW143315 and MW143325–MW143334. For phylogenetic comparisons, corresponding sequences of two ‘*Q. robertingeri,*’ five *Q. boulengeri*, one *Q. jiulongensis*, one *Q. exilispinosa*, one *Q. spinosa*, one *Q. shini*, one *Q. verrucospinosa*, one *Q. yei*, and one *Nanorana parkeri* were downloaded from GenBank (GenBank accession nos.: JN700886, KY441640, KC686711, KF199152, KX233867, KX233868, KX645665, KF199149, MH938690, MG820454, MK093237, KR087899, KJ700855, and KP317482).

**Table 1.** Genetic diversity and neutrality tests of *Quasipaa robertingeri*.

Pop ID	Locality	Latitude (° N)	Longitude (° E)	*n*	Haplotype	*h*	*π*	Tajima’s *D*	Fu’s *F*s
P1	Hejiang Co., Sichuan Prov.	28.6305	106.3004	21	H1–H5, H7, H9	0.667	0.00381	–0.60288	–1.017
P2	Jiangjin Dist., Chongqing City	28.6788	106.4176	11	H5, H7	0.600	0.00229	–1.05652	–0.290
P3	Chishui City, Guizhou Prov.	28.4499	105.9799	15	H6, H15, H16	0.448	0.00212	–1.68946	1.225
P4	Locality A, Xishui Co., Guizhou Prov.	28.4585	106.159	16	H10, H16	0.400	0.01259	1.29798	11.825*
P5	Locality B, Xishui Co., Guizhou Prov.	28.4664	106.3779	14	H7, H8, H9	0.670	0.00311	1.10915	2.093
P6	Locality A, Gulin Co., Sichuan Prov.	28.1861	105.7408	10	H15, H16	0.200	0.00037	–1.11173	–0.339
P7	Locality B, Gulin Co., Sichuan Prov.	28.2399	105.7517	23	H11H16	0.518	0.00135	–1.35873	–3.046
Lineage A	–	–	–	50	H1–H5, H7–H10	0.736	0.00443	–0.07680	–0.499
Lineage B	–	–	–	60	H6, H11H16	0.354	0.00111	–2.10347*	–3.658
Total	–	–	–	110	H1–H16	0.755	0.01771	1.11413	4.923*

*n*: number of samples; *h*: haplotype diversity; *π*: nucleotide diversity.

* *p* < .05.

Sequences were assembled and aligned using BioEdit 7.0.9.0 (Hall 1999) with default settings. Haplotypes were recognized using DnaSP 5 (Librado and Rozas 2009). Phylogenetic relationships of haplotypes of *Q. robertingeri* and its related species were reconstructed using maximum likelihood (ML), as implemented in the program PHYML 3.0 (Guindon et al. 2010). *Nanorana parkeri* was used as outgroup according to Che et al. (2009). For ML analyses, the best-fitting nucleotide substitution model was selected under the corrected Akaike information criterion (AICc) using JMODELTEST 2.1.7 (Guindon and Gascuel 2003; Darriba et al. 2012). The optimal nucleotide substitution model (GTR + I+G) was selected for the analyses. Non-parametric bootstrapping with heuristic searches of 1000 replicates was used to assess confidences of branches in ML trees. Pairwise uncorrected *p*-distances between clades and populations were estimated using MEGA 6 (Tamura et al. 2013). In addition, a haplotype network of *Q. robertingeri* was constructed using maximum parsimony method in TCS 1.21 (Clement et al. 2000).

Haplotype diversity (*h*) and nucleotide diversity (*π*) were estimated using DnaSP. Genetic signals of departure from neutrality or potential population expansion were estimated for populations using Tajima’s *D* (Tajima 1989) and Fu’ *Fs* (Fu 1997) statistics, estimated in DnaSP.

## Results

Alignment resulted in 504 base pair long sequences. Sixteen haplotypes were recognized from 110 individuals from seven populations of *Q. robertingeri* (Table 1). Phylogenetic analyses clustered the 16 haplotypes of *Q. robertingeri* into a clade (Figure 1(C,D)). Ten specimens of *Q. boulengeri* collected in this study were nested with five *Q. boulengeri* sequences and two sequences named as ‘*Q. robertingeri*’ downloaded from GenBank in a clade (Figure 1(C)). The *Q. robertingeri* clade, the *Q. boulengeri* clade, and one *Q. verrucospinosa* were clustered into a big clade (bootstrap support = 70%) separating from other congeners (Figure 1(C)) though the relationships of the three species in this clade were not resolved. The *Q. robertingeri* clade contains two divergent lineages, lineages A and B. Lineage A contained nine haplotypes (Haps 1–9), and lineage B contained seven haplotypes (Haps 10–16) (Figure 1(C)). Lineage A contained samples from populations P1, P2, P4, and P5, and lineage B contained samples from populations P3, P5, P6, and P7 (Figure 1(B)).

The least mean genetic distance between *Q. robertingeri* and its congeners was 11.5% (vs. *Q. boulengeri*). Genetic distance between lineages A and B of *Q. robertingeri* was 3.4%. Genetic distances between populations fall in the range of 0.0–4.8%, with an overall average at 2.3%.

Haplotype diversity (*h*) and nucleotide diversity (*π*) are presented in Table 1. The total haplotype diversity of *Q. robertingeri* was 0.755. Lineage A had higher haplotype diversity (0.736) than B (0.354). In populations, the population P4 showed the highest haplotype diversity (0.670), followed by the P1 population (0.667) and P2 (0.518), and other populations showed low haplotype diversity (*h* < 0.5). The total nucleotide diversity was 0.01771, but two major lineages have low nucleotide diversity (*π* < 0.045). The population P4 showed the highest nucleotide diversity (0.01259) and other populations exhibited low nucleotide diversity (*π* < 0.0039). In the total population and lineage A, Tajima’s *D*, and Fu’s *F*s tests all resulted in no-significant positive values (*p*>.05), and as well, all populations had no significant negative values (*p*>.05; Table 1), suggesting that there was no recent population expansion in these groups. As note, in lineage B, Tajima’s *D* was significant negative (*p*<.05; Table 1), indicating a signal for a recent expansion on population size of this lineage.

## Discussion

In this study, we explored the phylogenetic status and diversification of the mountain frog *Q. robertingeri* through sampling populations across its narrow distributional range in southwestern China (Figure 1(C)). Che et al. (2009) and Zhang et al. (2018) all based on sequences of a very little number of samples of ‘*Q. robertingeri*’ proposed that ‘*Q. robertingeri*’ was invalid. However, in our tree based on mitochondrial COI gene sequences showed that the samples recognized as ‘*Q. robertingeri*’ in Che et al. (2012) and Zhang et al. (2018) were nested into the *Q. boulengeri* clade, but the true *Q. robertingeri* samples was divergent from its closely related species *Q. boulengeri* and *Q. verrucospinosa* (Figure 1(D)). Moreover, the smallest genetic distance on COI gene was 11.5% between *Q. robertingeri* and *Q. boulengeri*, being much higher and near four times of 3% as interspecific genetic distance proposed by Zhang et al. (2018) and Vences et al. (2005). Hence, all COI sequences recognized as ‘*Q. robertingeri*’ on GenBank were misidentified, and *Q. robertingeri* should be a valid species. Obviously, *Q. robertingeri* and *Q. boulengeri* are sympatric in the distributional range of *Q. robertingeri* (Figure 1(C)), easily preventing the classifications of the two species also with the reason on the superficially morphological similarity of them. Our result indicated that to evaluate the validity of a species should first take a comprehensive sampling on population level of it and its closely related species.

All 110 samples of *Q. robertingeri* were clustered into two major divergent lineages (Figure 1(D,E)). They should represent long-time evolutionary units of biodiversity with mean genetic distance of 3.4%. But within each lineage, the divergence between haplotypes was shallow indicating that populations in each lineage were probably young. Neutrality tests suggested that lineage B has been experienced a recent population expansion. Accordingly, the locality for the population P4 located between the distributional ranges of lineages A and B might be the secondary contact area. Based on the results of this study, we at least need to protect each independent lineage of the endemic species as soon as possible to avoid losing important genetic diversity.

## Data Availability

The data of this study are openly available in figshare at https://doi.org/10.6084/m9.figshare.13153115.v1.
